# Time-dependent changes in genome-wide gene expression and post-transcriptional regulation across the post-death process in silkworm

**DOI:** 10.1093/dnares/dsae031

**Published:** 2024-11-15

**Authors:** Lin-Yu Yang, Da-Rui Tang, Shi-Qi Luo, Wei-Wei Li, Yu-Hang Jiang, Lian-Bing Lin, Qi-Lin Zhang

**Affiliations:** Faculty of Life Science and Technology, Kunming University of Science and Technology, Kunming 650500, China; Faculty of Life Science and Technology, Kunming University of Science and Technology, Kunming 650500, China; Faculty of Life Science and Technology, Kunming University of Science and Technology, Kunming 650500, China; Kunming Institute of Zoology, The Chinese Academy of Sciences, Kunming 650201, China; College of Food Science, Southwest University, Chongqing 400715, China; Faculty of Life Science and Technology, Kunming University of Science and Technology, Kunming 650500, China; Faculty of Life Science and Technology, Kunming University of Science and Technology, Kunming 650500, China

**Keywords:** post-mortem, silkworm, gene expression, miRNA, post-transcriptional regulation

## Abstract

Despite death marking the end of life, several gene expression and miRNA-mediated post-transcriptional regulation events may persist or be initiated. The silkworm (*Bombyx mori*) is a valuable model for exploring life processes, including death. In this study, we combined transcriptomics and miRNAomics analyses of young, old, and post-mortem silkworms across the entire process after death to unravel the dynamics of gene expression and miRNA-mediated post-transcriptional regulation. In total, 171 genes exhibited sustained differential expression in post-mortem silkworms compared to the pre-death state, which are primarily involved in nerve signalling, transport, and immune response. Post-mortem time-specific genes were associated with cell cycle regulation, thermogenesis, immunity, and zinc ion homeostasis. We found that the down-regulated expression of 36 genes related to transcription, epigenetic modification, and homeostasis resulted in a significant shift in global gene expression patterns at 2 h post-death. We also identified 5 mRNA-miRNA pairs (i.e. bmo-miR-2795-*mhca*, 2784-*achi*, 2762-*oa1*, 277-5p-*creb*, and 1000-*tcb1*) associated with stress hormone regulation, transcription activity, and signal transduction. The roles of these pairs were validated through *in vivo* experiments using miRNA mimics in silkworms. The findings provide valuable insights into the intricate mechanisms underlying the transcriptional and miRNA-mediated post-transcriptional regulation events in animals after death.

## 1. Introduction

After death, an organism loses the ability to acquire necessary substances for metabolism. In addition, there is a gradual depletion of the remaining limited substances, leading to a sudden reduction in the homeostasis of the internal environment. Consequently, harmful factors accumulate, triggering a step-wise shutdown of life activity after death until complete collapse.^1–3^ Post-mortem, individuals undergo a sequential series of changes: immediate and irreversible cessation of the vital functions in the brain, heart, and lungs; temperature dysregulation, muscle relaxation, and cessation of blood circulation occur during early post-mortem phases; and autolysis and putrefaction are detected in late post-mortem stages.^1–4^ These vital signs and phenotypic characteristics provide only a basic overview of the changes occurring in an organism post-mortem. The dynamics of transcription and gene expression changes and the corresponding post-transcriptional regulation by miRNAs throughout the entire post-mortem period, from the death until the complete degradation of the major biological macromolecules, such as total RNA as investigated in the current study, remain largely unexplored.

Limited studies have characterized the transcriptional profiles of post-mortem cells, tissues, and organs, revealing that transcriptional changes occur in response to the stress induced by death.^1,4,5^ For instance, genes associated with stress, immune response, and substance transport (such as *hsp1*, *il1b*, and *tnpo1*) are significantly upregulated in post-mortem zebrafish and mice to compensate for lost homeostasis.^1^ Active and ongoing transcriptional regulation and a cascade of transcriptional events exist in multiple tissues of post-mortem donors to manage death.^4^ A time-dependent increase in gene expression of astrocytes and microglia in the human brain at 24 h post-death (hpd) prevents rapid loss of neuronal activity.^5^ However, these investigations are limited to the use of partial tissues of individuals to explore transcriptional change post-mortem, as it is challenging to collect samples from the entire body of experimental animals with medium to large body sizes. This limitation may lead to bias in comprehensively understanding death events from the perspectives of the whole body. miRNAs, a class of endogenous non-coding single-stranded RNA molecules that inhibit gene expression, typically bind to the 3ʹ untranslated region (UTR) of their target genes, playing critical biological roles in responding to stress and maintaining homeostasis.^6,7^ However, the mechanisms underlying miRNA-mediated post-transcriptional regulation in post-mortem organisms remain entirely unexplored.

Silkworm (*Bombyx mori*) has several genes that are homologous to human disease-causing genes, as well as organ and tissue composition similar to humans.^8–10^ In recent years, numerous studies have used silkworms as experimental models to explore the transcriptional and post-transcriptional regulatory mechanisms underlying human life activities and disease occurrence.^9,11,12^ Therefore, the exploration of transcription and transcriptional regulation in silkworms provides valuable insights into human life activities. Moreover, silkworms experiencing natural death are a suitable model for investigations related to the natural death of animals due to less interference compared to animals subjected to unnatural death causes, such as artificial stresses, as observed in zebrafish and mice that undergo sudden euthanasia.^1^ In addition, the suitable body size of silkworms facilitates the exploration of individual-level death events across the complete post-mortem period. In summary, silkworms are valuable for studying the dynamics of global transcription and miRNA post-transcriptional regulation in post-mortem animals.

In this study, we systemically collected adult silkworm samples at 8 time points, including 2 sampling points during the living stage (young and old age), and the other time points covering the entire post-mortem stage. Transcriptome and miRNAome data were generated by sequencing these samples. We identified differentially expressed genes (DEGs), time-specific genes (TSGs), dynamical network biomarker (DNB) genes, and mRNA-miRNA pairs specific to post-mortem stages. Moreover, we conducted a functional enrichment analysis of these genes. miRNA targets obtained through the prediction of mRNA-miRNA pairs were validated through *in vivo* experiments by injecting agomir and antagomir miRNA mimics to silkworms.

The primary motivation for this study was driven by curiosity in the processes involved in the shutting down of a complex biological system—which has received little attention to date, as a similar reason presented in.^1^ Other fields of research have examined the shutdown of complex systems (e.g. societies^13^ and government^14^). To the best of our knowledge, however, no study has examined post-mortem dynamics of transcripts from invertebrates, particularly miRNA-mediated post-transcriptional regulation. In addition, shutdown and further collapse of a complex biological system require time and energy and could provide new insights into interesting pathways.^1^ Hence, the transcripts, including mRNAs and miRNAs, are involved in day-to-day survival as well as stress compensation.^1^ However, since death means the end of life—one would expect mRNAs will change with time after death. How the transcription and therein miRNA-mediated post-transcriptional regulation dynamically change with post-mortem time was the goal of this study. This work will enrich the understanding of changes in transcriptomic profiles after invertebrate death, providing a more comprehensive knowledge for the occurrence of a step-wise shutdown in animal death.

## 2. Materials and methods

### 2.1. Collection of silkworm samples

**Fig. 1.**
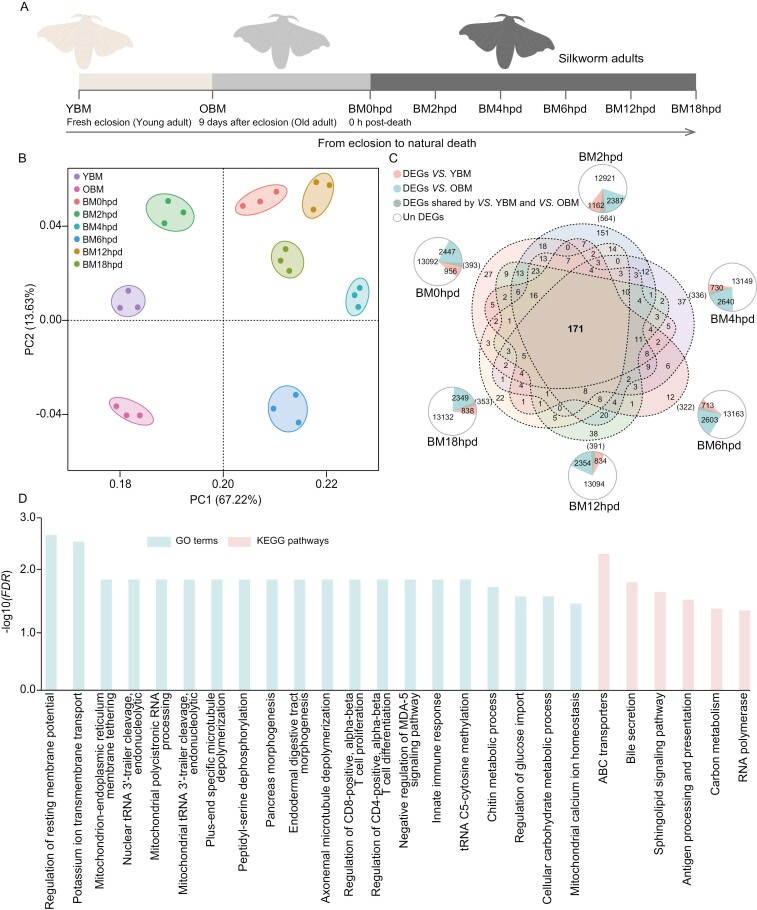
DEGs in adult silkworm post-mortem. a) A flow diagram illustrating the sampling process. YBM freshly emerged from pupae. OBM: old silkworm. BM0hpd, BM2hpd, BM4hpd, BM6hpd, BM12hpd, and BM18hpd represent silkworm collected post-mortem at 0, 2, 4, 6, 12, and 18 h post-death, respectively. b) PCA plot showing the clustering of the RNA-seq libraries from the different stages. c) Summary of the number of DEGs across the various stages. The area of the sector in the circle represents the number of genes. d) Enriched GO terms (light green) and KEGG pathways (pale pink) significantly (*FDR* < 0.05) associated with the CPRGs.

Healthy pupae of *B. mori* (a hybrid strain of the domesticated silkworm Qiufeng × Baiyu) were obtained from Babei Apparels Co., Ltd, Shengzhou, Zhejiang Province, China and reared in an incubator (CZX-250 BS-III, Shanghai CIMO Medical Instrument Co., Ltd) at room temperature, with 70% relative humidity and a 12/12 h photoperiod in the Faculty of Life Science and Technology, Kunming University of Science and Technology, Kunming, China, as described previously.^15^ The experimental silkworms ceased feeding from eclosion until natural death. Typically, naturally deceased silkworms experience approximately 10 d from eclosion to death (adult stage).^8,16^ In this study, the silkworms reached the adult stage and survived for 10 d before their natural death. Adult silkworm and their deceased bodies at different stages were systematically collected until the samples (after 18 hpd) were no longer available for extraction of high-quality total RNA. These specimens were assigned into 8 groups based on the specific developmental stages and different post-mortem intervals. Young adult silkworms emerging from pupae were randomly collected as young *B. mori* (YBM) samples. Adult silkworms typically exhibit significantly reduced movement activity than young individuals after 8–9 d of eclosion. Therefore, silkworm adults randomly collected on the ninth-day post-eclosion were designated as old *B. mori* (OBM). Adult silkworms were monitored every half hour from the ninth day after eclosion due to their impending death. Those showing no movement or response when touched on their head, chest, back, and abdomen with an insect needle were recorded as deceased, as described in a previous study with minor modifications.^17^ Fresh post-mortem samples were randomly collected as the BM0hpd group. Subsequent collections were made at 2, 4, 6, 12, and 18 h post-death (hpd), categorized as BM2hpd, BM4hpd, BM6hpd, BM12hpd, and BM18hpd groups, respectively. The experiments were conducted in 3 independent replicates to serve as biological replicates. Sixty silkworm adult individuals were randomly sampled at each time point, and immediately stored in batches at a -80 °C refrigerator (Thermo Scientific, USA). A flowchart illustrating the sample collection process is presented in Fig. 1a.

### 2.2. RNA isolation and sequencing

For total RNA isolation, each silkworm was homogenized separately using Trizol reagent (Invitrogen, USA), followed by treatment with DNaseI to eliminate genomic DNA impurities (Qiagen, Germany). The concentration of total RNA was assessed to ensure it exceeded >250 ng/mL, whereas its integrity was evaluated by a RIN value > 7, and its quality was confirmed by a 260/280 nm wavelength ratio between 1.8 and 2.0 using a NanoDrop 1000 spectrophotometer (Thermo Scientific). The quality RNA from 20 silkworms collected at each time point was adjusted to a uniform concentration of 200 ng/μL and pooled equally for sequencing, as previously described.^18^ These experiments were conducted as 3 independent replicates to serve as biological replicates. In total, the preparation of 24 mRNA-seq libraries (comprising 3 biological replications across 8 time points) and 16 miRNA-seq libraries (comprising 2 biological replications across 8 time points) was performed following the standard procedure at Beijing Genomics Institute (BGI, China; https://www.bgi.com/global). Briefly, mRNA was isolated according to the poly-A selection method by oligo(dT) beads and fragmented by buffer. Then, cDNA was synthesized using random hexamer primers. The resulting cDNA was subjected to end-repair, with ‘A’ base addition. Next, cDNA target fragments in size of 200–300 bp were selected on 2% agarose, followed by PCR amplification and quantification. For preparation of miRNA sequencing libraries, small RNAs in size of 18–30 nt were selected using polypropylene acyl amine gel electrophoresis (PAGE). Then, 5-adenylated, 3-blocked single-stranded DNA adapters were linked to the 3ʹ-end of the small RNAs, and 5ʹ-termini were linked to 5ʹ adaptors. Next, cDNA was synthesized via reverse extension of the RT primer, followed by PCR amplification and quantification. Quality of all the libraries was assessed on the Agilent Bioanalyzer 2100 system using DNA High Sensitivity Chips. Subsequently, these libraries were sequenced on a DNBSEQ sequencing platform using standard procedures at the BGI.

### 2.3. Preprocessing of mRNA-seq and miRNA-seq data, identification of miRNAs, and expression analysis

Quality control of the raw reads from mRNA-seq data involved filtering out sequences with adaptors, low-quality reads with >10% unknown bases, and sequences with more than 50% low-quality bases (*Q* value ≤ 10) using the SOAPnuke (v1.5.2) software with default parameters.^19^ The filtered reads were aligned to the silkworm reference genome (NCBI genome database: https://www.ncbi.nlm.nih.gov/genome/, version: Bmori_2016v1.0, 17,047 genes) using HISAT2 tool.^20^ Gene expression levels were quantified as fragments per kilobase of transcript per million mapped reads (FPKM) using RSEM (v1.3.0).^21^ Genes with FPKM ≥ 0.1 across all samples were used for downstream analysis.^22^ The reproducibility among the 3 biological replicates was assessed using principal component analysis (PCA) based on FPKM values. All genes from the silkworm genome were functionally annotated by searching against the NCBI non-redundant protein sequence (Nr), Gene Ontology (GO), and Kyoto Encyclopedia of Genes and Genomes (KEGG) databases.

The Trimmomatic (version 0.32) was used to eliminate low-quality sequences and adaptors for raw tags generated by miRNA-seq. Clean tags, ranging from 18 to 30 bp in length, were mapped to the reference genome and aligned to sequences deposited in the GenBank and Rfam databases to functionally annotate small RNAs.^23^ Non-miRNA small RNA tags, such as rRNA, scRNA, snoRNA, snRNA, and tRNA, were eliminated, and the remaining tags were used for downstream analysis. Subsequently, clean tags without highly repetitive sequences were obtained by filtering using RepeatMasker software (http://www.repeatmasker.org/). The clean tags were then queried against the miRBase 16.0 database using the Blastn tool to identify known miRNAs, with strict criteria of zero mismatches, as described in previous studies.^24,25^ miRNAs detected in at least two biological repeats at any one-time point were retained for analysis. The expression levels of miRNAs were quantified using transcripts per million (TPM).^25^ Subsequently, miRNAs with average TPM values less than 1 across all the samples were excluded.^18^ Correlation among samples was calculated using the corrr R package based on TPM values of miRNAs to assess the consistency between biological replicates.

### 2.4. Identification of DEGs and miRNAs (DEMs)

The expression levels of mRNAs and miRNAs in the YBM and OBM groups, which served as controls, were separately compared to the BM0hpd, BM2hpd, BM4hpd, BM6hpd, BM12hpd, and BM18hpd groups to identify DEGs and differentially expressed miRNAs (DEMs) using the DESeq2 package (v1.43.5),^26^ an R package suitable for DEG analysis of biological replicates fewer than 12 in RNA-seq.^27^ The resulting *P* values were adjusted using the Benjamini–Hochberg adjustment method to control the false discovery rate (*FDR*) of the significance test. The criteria for identifying DEGs and DEMs were |Log2 fold change| > 1 with *FDR* < 0.05.

### 2.5. Analysis of TSGs

The Mfuzz R package (v2.58.0) was used for temporal soft-clustering analysis of gene expression data across the eight stages to identify TSGs at each post-death time point.^28^ The expression levels of all mRNAs were normalized under default parameters. The normalized gene expression data were subjected to fuzzy c-means clustering using the mestimate function, with the optimal number of clusters set to 36 and the fuzzifier m parameter set to 1, as described previously.^29,30^ Subsequently, membership scores between mRNAs were estimated, and clusters containing genes with consistently specific expression patterns at each time point were identified as TSGs.

### 2.6. Analysis of DNB genes

The presence of a tipping point in gene expression indicates a sudden alteration in biological processes, where the genes initiating this key transition were considered potential DNB.^29,31,32^ The DNB approach was used to identify critical transition stages caused by gene expression changes in silkworms after death, aiming to identify DNB genes associated with the emergence of these critical transition points. The nonlinear dynamic theory indicates that a set of dominant genes (i.e. DNB genes) meeting the following 3 criteria denote the presence of a tipping point in gene expressed following the death of silkworms: (i) a significant increase in standard deviations of the expression of genes in this dominant group, (ii) a notable increase in Pearson correlation coefficients among genes (expression levels) in this dominant group, (iii) and significant decrease in Pearson correlation coefficients of genes between this group and other groups.^29,31,32^ Several sets of candidate DNB genes were identified using a slightly modified DNB method, adapted from a previous study^32^ and incorporated the 3 criteria described above. Each set of candidate DNB genes was evaluated to determine their potential as indicators of critical transition points in silkworm after death by calculating the average standard deviation and the average correlation strength of gene expression levels in each set.^32^ A time point was identified as a critical transition point if both the average standard deviation and average correlation strength of gene expression levels in a set of candidate DNB genes peaked simultaneously, and the DNB genes were determined. Analysis of DNB genes was performed using the hclust function in R software.^33^

### 2.7. Mutilple criteria identification of target genes of miRNAs

Target genes of miRNAs were identified using a multiple-criteria method, as described previously.^18^ The process involved assessing credible mRNA-miRNA pairs based on sequence complementarity and correlation of expression levels between mRNAs and miRNAs.

The MiRanda^34^ and RNAhybrid^35^ tools were used to predict the target genes of miRNAs based on sequence complementary between mRNAs and miRNAs. The results generated by these tools were intersected for downstream analyses. In addition, the expression levels of the mRNAs and miRNAs across the 8 time points were analysed using the weighted correlation network analysis (WGCNA) R package to generate co-expression modules containing mRNAs and miRNAs.^36,37^ Genes significantly correlated in their expression level (*P* values < 0.05) were grouped into sets based on the Pearson correlation coefficients between every 2 RNA molecules (mRNA-miRNAs, mRNA-mRNAs, and miRNA-miRNAs) were used to construct a similarity expression gene matrix. Subsequently, an adjacency matrix was derived from the similarity matrix of gene expression to calculate the topological overlap measure (TOM), indicating the distance among genes. A topological matrix was then obtained through transformation of the adjacency matrix. A clustering dendrogram of the topological matrix was constructed by separating gene modules based on co-expression and merging the modules with similar expression patterns based on TOM dissimilarity. Each module comprised a combination of miRNAs and mRNAs, forming mRNA-miRNA pairs with significant correlations (*P* values < 0.05). Furthermore, Spearman’s rank correlation between all the mRNAs and miRNAs was calculated using the cor.test function in the R statistical package to validate the WGCNA results. mRNA-miRNA pairs with an absolute value of correlation coefficient > 0.8 and *P* values < 0.05 were selected for subsequent analysis. Notably, miRNAs typically bind to sites in the mRNA 3ʹUTR to downregulate gene expression, resulting in a negative correlation between miRNA abundance and the expression level of target genes.^6,7^ Therefore, mRNA-miRNA pairs with a negative coefficient < − 0.8 were retained for further analysis.

The final targets of miRNAs were determined by intersecting the results obtained from the two sequence-matching methods and the two gene expression methods. Moreover, if both members of an mRNA-miRNA pair were detected in the list of DEGs and DEMs, the pair was considered to be involved in the post-death process of silkworm.

### 2.8. Functional enrichment analysis

GO and KEGG enrichment analysis was conducted using Blast2GO pipeline^38^ and KOBAS 2.0^39^ tool with default parameters. Fisher’s exact test was used to calculate the significance levels (*P* values) of the GO and KEGG terms, which were further adjusted for *FDR* using the Benjamini–Hochberg method. GO and KEGG terms with *FDR* < 0.05 were considered significant.

### 2.9. *In vivo* determination of mRNA-miRNA relationship

Two mRNA-miRNA pairs were chosen randomly to verify miRNA regulation on the corresponding target genes through *in vivo* experiments using agomir and antagomir mimics of miRNAs in silkworms, following methodologies described in previous studies.^40,41^ Agomir, antagomir, and negative control (N.C.) of bmo-miR-277-5p and bmo-miR-2762 were synthesized by Sangon Biotechnological Co. (Shanghai, China). Twenty adult silkworms were randomly selected, and 15 μg of miRNA agomir mimics were administered in each individual by injection into the ventral abdomen using a microsyringe (Shanghai Bolige Industry & Trade Co., Ltd, China).^40^ Similarly, antagomir mimics and N.C. mimics of miRNAs were separately injected into silkworms (*n* = 20 individuals). The experimental silkworms were reared in an incubator (CZX-250 BS-III, ShangHai CIMO Medical Instrument Co., Ltd) with the conditions maintained at 27 °C ± 2 °C, 70% relative humidity, and a 12.12 h photoperiod for 2 d. Subsequently, the experimental samples were collected to test the physiological parameters.

As target genes of bmo-miR-2762 and bmo-miR-277-5p, respectively, octopamine receptor beta1 (*oa1*) promotes the release of intracellular cyclic adenosine monophosphate (cAMP) and Ca^2^^+^^,42^ and increased expression of the gene (*creb*) that encoded the cAMP-responsive element binding protein promotes the production of cAMP and Ca^2^^+^.^43^ Therefore, the content of cAMP and Ca^2^^+^ significantly positively correlated with the expression levels of these 2 genes, but is opposite to that of the miRNAs. Here, the focus is thus placed on the levels of cAMP and Ca^2^^+^ to determine of mRNA-miRNA relationship above predicted by bioinformatics. First, cAMP and Ca^2^^+^ were separately detected using the relevant capture antibodies. Subsequently, horse radish peroxidase (HRP)-labelled detection antibodies were used to bind the capture antibodies, followed by visualization through 3,3ʹ,5,5ʹ-tetramethylbenzidine staining of HRP. This process was conducted according to the manufacturer’s protocols for insect cAMP (TOPEL30261, Biotopped, Beijing, China) and Ca^2^^+^ (TOPEL30260) ELISA kit, using a one-step double antibody sandwich enzyme-linked immunosorbent assay. The absorption values of the samples were measured using a multimode Varioskan LUX fluorescence microplate reader (Thermo Scientific) at a wavelength of 450 nm. The experiments were performed as 3 independent replicates to represent three biological replicates.

### 2.10. Statistics analysis

The data were presented as the mean of biological replicates ± S.E. Statistical significance in comparisons was determined using one-way ANOVA followed by the least significant difference (LSD) test in IBM SPSS Statistics (V22.0), unless stated otherwise. Differences among groups were considered significant at a *P* value < 0.05.

## 3. Results

### 3.1. Overview of mRNA-seq and miRNA-seq data

MRNA-seq (Supplementary Table S1) and the corresponding miRNA-seq (Supplementary Table S2) data of 40 sequencing libraries from adult silkworm samples collected at 8 time points at young, old, and post-mortem stages were generated. The average raw reads was approximately 45 million for the mRNA-seq, with the proportion of clean reads accounting for 97% of the total reads. The Q20 and Q30 values of mRNA-seq data beyond 96% and 91%, respectively. In addition, an average value of approximately 30.23 million raw tags were obtained in miRNA-seq, with 96.81% of these identified as clean tags. The average genome mapping rates of mRNA-seq and miRNA-seq data were 77.33% and 82.17%, respectively, indicating the reliability of the data for the subsequent analyses.

Known miRNAs (ranging from 258 to 305) were detected in all the miRNA-seq libraries (Supplementary Table S2). The mature miRNAs with a size of 22 nt showed the highest abundance, followed by miRNAs with 28 and 23 nt (Supplementary Table S3). The length distribution of identified miRNAs was consistent with findings in silkworm and other metazoans.^18,44^

PCA analysis of mRNA-seq revealed 8 distinct clusters, demonstrating evident separation among different groups (Fig. 1b). Correlation analysis miRNA expression exhibited a significantly higher correlation between samples from the same time point compared to those from different points (Supplementary Fig. S1). These results indicate reliable treatment of the experimental samples and RNA-seq analysis.

### 3.2. Identification and functional analysis of DEGs and DEMs

In this study, 5,080 genes were identified as DEGs between at least one stage before or after death (Supplementary Table S4), with 171 of these genes shared among all the comparison groups and identified as core post-mortem-related genes (CPRGs) (Fig. 1c and Supplementary Table S5). The CPRGs were significantly (*FDR* < 0.05) associated with 20 enriched GO terms and 6 enriched KEGG pathways, primarily involved in nerve function, transport, immune response, and transcription (Fig. 1d). In addition, 143 DEMs were obtained in this study (Supplementary Table S6).

### 3.3. Identification and functional analysis of TSGs

**Fig. 2.**
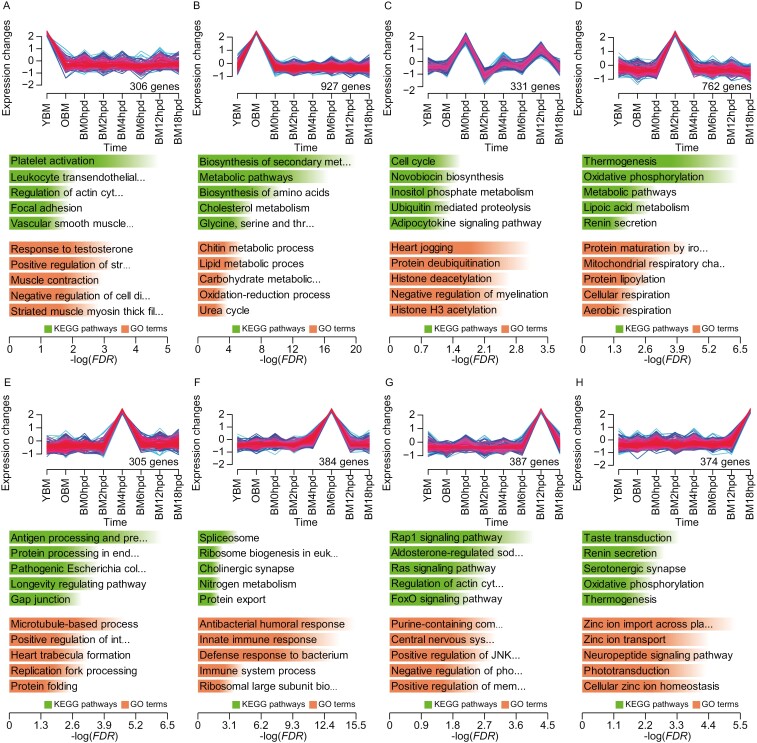
TSGs in silkworm. Time-specific gene clusters at YBM a), OBM b), BM0hpd c), BM2hpd d), BM4hpd e), BM6hpd f), BM12hpd g), and BM18hpd h), respectively. The number of genes assigned to each cluster is presented in the lower right position in each coordinate. Histograms indicate the top 5 significantly enriched KEGG pathways and GO terms, with significance levels presented as green and orange bars, respectively.

All genes were categorized into 36 gene expression clusters through Mfuzz analysis (Supplementary Fig. S2), with 8 expression clusters exhibiting distinct time-specific upregulation (Fig. 2), comprising 3,415 TSGs (Supplementary Table S7). The highest number of TSGs was observed at OBM (927), followed by BM4hpd (402), BM12hpd (386), BM6hpd (384), BM18hpd (374), BM0hpd (331), YBM (306), and BM2hpd (305). The enrichment analysis revealed the highest number of KEGG pathways significantly enriched by TSGs at BM12hpd (22), followed by OBM (20), BM18hpd (14), YBM (13), BM4hpd (11), BM2hpd (7), BM0hpd (5), and BM6hpd (5) (Supplementary Table S8). Moreover, the number of GO terms significantly enriched by TSGs was maximum at OBM (20), followed by BM12hpd (18), BM18hpd (17), YBM (16), BM0hpd (15), BM4hpd (15), BM2hpd (14), and BM6hpd (13) (Supplementary Table S9). TSGs identified at YBM were primarily implicated in immune response, muscle contraction, and cell division (Fig. 2a). TSGs identified at OBM were predominantly associated with chitin metabolism, biosynthesis and metabolism of amino acids, and oxidation–reduction process (Fig. 2b). At BM0hpd, TSGs were primarily involved in the cell cycle, response to hypoxia, and heartbeat (Fig. 2c). TSGs identified at BM2hpd were mainly associated with thermogenesis, protein modification, and respiration (Fig. 2d). At BM4hpd, TSGs were predominantly involved in protein processing, pathogenic infection, and longevity regulation (Fig. 2e). At BM6hpd, TSGs were mainly associated with spliceosome, antibacterial immunity, and nervous system (Fig. 2f). TSGs identified at BM12hpd were primarily implicated in signal transduction, development, and reabsorption of substances (Fig. 2g). The TSGs identified at BM18hpd were implicated in taste transduction, zinc ion metabolism, and nerve signalling (Fig. 2h).

### 3.4. Identification and functional analysis of DNB genes

**Fig. 3.**
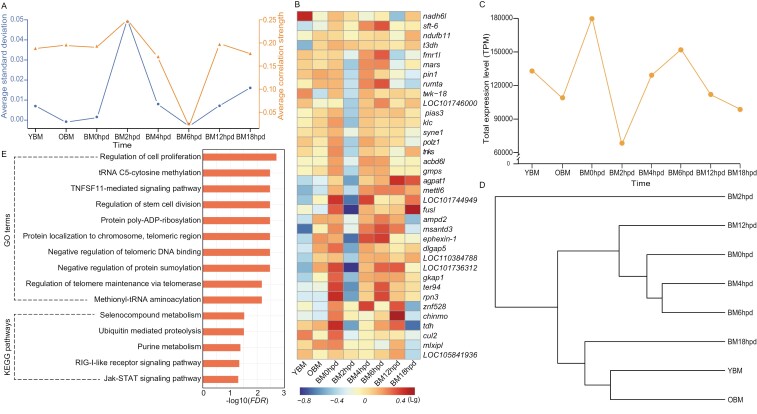
DNB genes. a) Critical transition events for global gene expression. b) A heatmap of DNB genes. c) Line chart and d) cluster dendrogram of the total expression level TPM of the DNB genes across time points. e) Enriched GO terms and KEGG pathways associated with the DNB genes, with significance levels presented as orange bars. Detailed information of the DNB genes is presented in Supplementary Table S10.

DNB analysis revealed a significant increase in gene expression at 2 hpd in silkworms (Fig. 3a). Consequently, the corresponding 36 DNB genes were identified (Supplementary Table S10), with most of them exhibiting down-regulated expression at BM2hpd compared to other time points (Fig. 3b). Notably, minimum expression levels of these genes were observed at BM2hpd (Fig. 3c). The cluster tree illustrated that the overall expression pattern of the DNB genes differed significantly at 2 hpd compared with the other time points (Fig. 3d). These DNB genes were associated with significant enrichment (*FDR* < 0.05) of 10 GO terms and 5 KEGG pathways, primarily involved in cell proliferation, selenocompound metabolism, methylation, proteolysis, and signal transduction (Fig. 3e).

### 3.5. Integrated analysis of RNA-seq and miRNA-seq

**Fig. 4.**
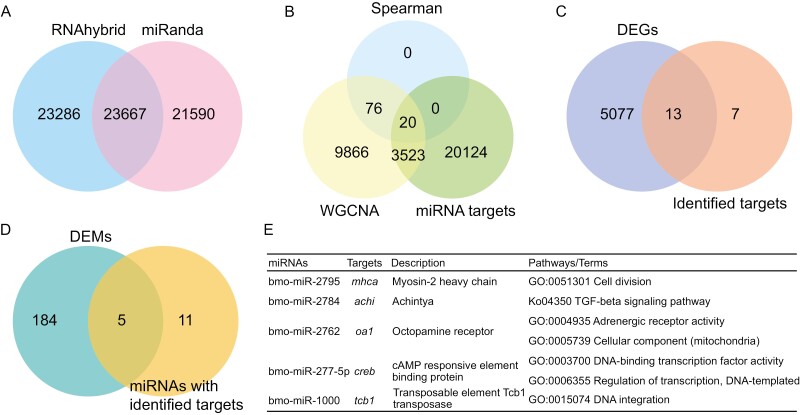
Functional annotation of target genes of miRNAs. a) A Venn diagram of the number of targets common between RNAhybrid and miRanda tools based on sequence-matching methods. b) A Venn diagram of the number of the targets common for the sequence matching and expression correlation (WGCNA and Spearman correlation analysis) methods. A Venn diagram of the number of DEGs c) and DEMs d) shared by the members of the mRNA-miRNA pairs identified through the above methods. e) A summary of the identified mRNA-miRNA pairs.

In total, 46,953 and 45,257 target genes of miRNA were identified using RNAhybrid and Miranda tools, respectively (Fig. 4a). Among these, 23,667 genes were common for the 2 algorithms. Therefore, they were retained as the miRNA targets based on sequence-matching methods. In addition, 66 co-expression modules of mRNAs-miRNAs were identified through WGCNA analysis (Supplementary Fig. S3), and resulting in the identification of 13,485 target genes. Spearman’s rank correlation analyses indicated that 96 targets exhibited significantly negative correlation (*P* values < 0.05 and coefficient values < − 0.8) with miRNAs at the expression level. In summary, 96 genes common in the 2 algorithms were identified as miRNA targets based on the expression correlation methods (Fig. 4b).

A total of 20 genes (distributed in 20 mRNA-miRNA pairs and regulated by 16 miRNAs) were identified through sequence matching and expression level-based analyses (Fig. 4b, Supplementary Table S11). Notably, 13 of these 20 targets were identified as DEGs (Fig. 4c, Supplementary Table S12), whereas 5 of the 16 miRNAs were DEMs (Fig. 4d, Supplementary Table S13). Among the identified pairs, 5 mRNA-miRNA pairs were finally retained as final targets (Fig. 4E). These targets were primarily associated with cell motility, TGF-beta signalling pathway, adrenergic receptor activity, and DNA-binding transcription factor activity.

### 3.6. *In vivo* validation

**Fig. 5.**
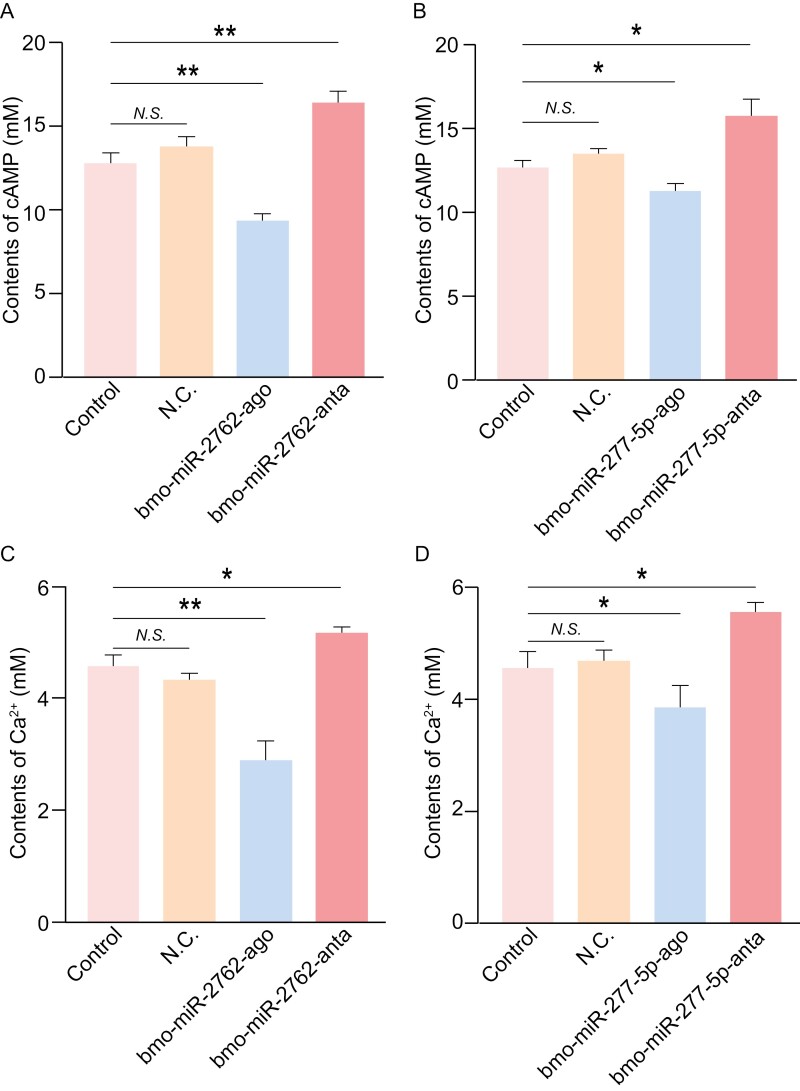
The levels of cAMP and Ca^2^^+^ in adult silkworm injected with bmo-miR-2762-agomir and bmo-miR-2762-antagomir as well as bmo-miR-277-5p-agomir and bmo-miR-277-5p-antagomir. a) and b) Contents of cAMP. c) and d) Ca^2^^+^ levels. All data are presented as mean ± S.E. (*n* = 3). Significance values were calculated for each group, compared to the controls, using one-way ANOVA followed by the least significant difference test. *P < 0.05, **P < 0.01, and *N.S.* denotes no significance.

cAMP levels were significantly lower in adult silkworms injected with bmo-miR-2762 or bmo-miR-277-5p agomir (*P* values < 0.05) than the N.C. and control groups at 48 h post-injection. Notably, the content of cAMP showed no significant difference between the 2 control groups (Fig. 5a and b). Conversely, the content of cAMP was significantly higher in silkworm groups injected with bmo-miR-2762 or bmo-miR-277-5p antagomir (*P* values < 0.05) compared to agomir-treated, N.C. and control groups. The content of Ca^2^^+^ presented a similar pattern across silkworm groups injected with controls, agomir, and antagomir of bmo-miR-2762 or bmo-miR-277-5p (Fig. 5c and d).

## 4. Discussion

In this study, several genes involved in the regulation of neuronal activity (such as *sf3b1*, *msh-a*, and *cart*), transmembrane transport of potassium ions (including *twk18*, *nalcn*, and *slc9a3*), and cellular potassium ion homeostasis (*kcnj2*) were identified in the list of CPRGs. Two terms, including regulation of the resting membrane potential and transmembrane transport of potassium ions, were significantly enriched by CPRGs. Epigenetic regulation is implicated in neural activity in the post-mortem human brain.^45^ In the current study, we observed that CPRGs were associated with significant enrichment of tRNA C5-cytosine methylation. These results indicate that neural activity in post-mortem silkworms is facilitated by the stabilization of membrane potential, which is mediated by homeostasis of potassium ions and methylation processes. Moreover, for a certain duration in post-mortem, several neural cells still possess activity of the resting membrane potential^46^ and time-dependent expression changes in the human brain.^5^ All the neural activity started with a change in the resting membrane potential, which is primarily determined by selective permeability of potassium channel on membrane of neurone.^47^ These findings suggest that neural activity resulting from potassium ions-mediated activity of membrane potential and epigenetic changes may be a conserved phenomenon in post-mortem Bilateria.

Transport-related genes are involved in compensating for metabolic dysregulation in post-mortem by affecting the efficiency of transmembrane exchange of carbohydrates and ions.^1^ In this study, several transport- and transmembrane-related genes were identified as CPRGs, such as *abc*, *slc25a29*, and *oct*, with the ABC transporters pathway significantly enriched by CPRGs. Furthermore, CPRGs associated with respiration and mitochondrial electron transport (such as *me1* and *ndu1*), and ion transport and carbohydrate metabolism (*wh3*) were identified in this study. These genes were associated with the enrichment of mitochondrial calcium ion homeostasis, cellular carbohydrate metabolic process, and regulation of glucose import. These findings indicate that transport-related genes are involved in restoring metabolic homeostasis in post-mortem silkworm.^1^ Interestingly, expression changes of genes related to mitochondrial electron transport were also detected in post-mortem human brain,^48^ showing key roles of the altered energy production efficiency in animals after death. In addition, several CPRGs (such as *cec1*, *rpc8*, and *hsp68*) and their enriched functional terms (including antigen processing and presentation) were identified, which are involved in immune response as reported in post-mortem human, zebrafish, and mice.^1,49^ In insects, the cuticle is the first protective barrier against invasion by exogenous substances, maintaining the integrity of the internal environment.^50^ In this study, we identified 7 CPRGs that encoded cuticular proteins (including *cpr78*, *cpr5*, and *cpr23*), which were associated with significant enrichment of the chitin metabolic process. These findings indicate that defensive responses are significantly affected in silkworms following death. Therefore, changes in immune and defensive response were important biological processes in post-mortem animals, which are not limited to living life.

Gene expression generally decreases after death. However, upregulated/active genes post-mortem are particularly interesting and important. This study focussed on the upregulated TSGs identified post-mortem. Cell cycle regulation-related genes promote cell division to repair abnormal cells in human post-mortem tissues.^51,52^ The cell cycle phases, such as mitosis, are regulated by histone modifications.^53^ In this study, several TSGs involved in cell cycle and histone structural modification, such as *cdk2*, *ccn3*, and *cdk7*, were identified at 0 hpd. Notably, these genes were associated with enriched terms implicated in histone modification, indicating that cell repair regulated by histone modification occurred in post-mortem silkworms, and cell cycle regulation implemented by histone modifications is a conservative mechanism for promoting the self repair of cells between silkworm and human after death. *Nipblb* and *foxj1a*, key genes that regulate heart jogging, were identified as TSGs at 0 hpd. These genes are negative feedback signals for cardiorespiratory dysfunction caused by death,^1^ resulting from the absence of oxygen uptake ability in newly deceased silkworms. Moreover, *elob*, which mediates the typical hypoxia response,^54^ was identified as a TSG at 0 hpd. Similarly, upregulation of several genes associated with cellular responses to hypoxia was also found in human after death.^51^ At 2 hpd, TSGs were associated with significant enrichment of thermogenesis, energy metabolism, and protein lipoylation (which stabilizes TCA cycle).^55^ Moreover, the increasing abundance of metabolites (e.g. long-chain acylcarnitines) involved in mitochondrial energy metabolism was detected in human after death,^56^ indicating the presence of a concentrated energetic response in both post-mortem silkworm and human. At 4 and 6 hpd, TSGs were predominantly associated with a notable enrichment of immunity and DNA repair, indicating a dominant role of immune response and repair mechanisms in this post-mortem period. Interestingly, time-specific expression of longevity-related genes (including *hsf1*, *l(2)efl*, and *hspa1a*) and longevity regulating pathway was detected at 4 hpd, implying that lifespan regulation mechanisms may still be active after death. This is the first report in animals, providing novel insights into roles of longevity-related genes.

At 12 hpd, *foxg1*, a transcription factor that regulates postmitotic cortical neurone specification,^57^ was identified as an activated TSG. Notably, functions related to neural signal transduction, such as Rap1, FoxO, and Ras signalling pathways, were significantly enriched. Furthermore, TSGs involved in the regulation of the actin cytoskeleton, involved in modulating signal output and intensity,^58^ were enriched. This finding indicates the integration and activity of neural signal transduction in post-mortem silkworms. Zinc ion homeostasis is crucial for neural activity/excitation and ATP production.^59,60^ Pathways associated with zinc homeostasis (including import and transport mechanisms) were significantly enriched at 18 hpd. Notably, *htr*, a TSG that encodes 5-hydroxytryptamine receptor, as a prominent excitatory transmission receptor, was identified. This TSG was associated with enrichment of excitatory transmission-related functions including neuropeptide signalling pathway and serotonergic synapse. In addition, several genes (including *atplb*, *atp5b*, and *acly*) that encode ATP synthetase exhibited time-specific upregulation at 18 hpd and were associated with the enrichment of oxidative phosphorylation and thermogenesis. These findings imply that neural excitation and ATP production are maintained through zinc ion homeostasis in post-mortem silkworm. This potential mechanism is first reported here, likely presenting a lineage-specific mechanism of neural activity and energy production in post-mortem insects.

A significant shift in global gene expression triggered by changes in the expression of 36 DNB genes was observed at 2 hpd in post-mortem silkworms. Previous studies reported that DNB genes drive changes across the entire organism.^29,31,32^ This phenomenon may be attributed to the onset of extensive physiological collapse in the silkworm body at 2 hpd. Transcription factors and transcription-related proteins^61^ are common targets of sumoylation.^62^ In the current study, we identified several down-regulated DNB genes involved in the regulation of transcription factor activity (such as *znf528*, *mlxipl*, and *pias3*) in silkworms after death. Moreover, DNB genes were associated with enrichment of negative regulation of protein sumoylation. These findings suggest a widespread suppression of transcription factor activity in post-mortem silkworms. In addition to transcriptional regulation, epigenetic modification modulates cell differentiation and proliferation.^63,64^ In this study, DNB genes were associated with the enrichment of several GO terms related to cell proliferation and division as well as methylation, indicating a decrease in cell proliferation and differentiation. Methionine-tRNA ligase catalyses selenocompound metabolism to enhance antioxidant defense.^65^ The gene *mars*, which encodes methionine—tRNA ligase, was identified as a down-regulated DNB gene. Moreover, the selenocompound metabolism pathway was significantly enriched by DNB genes, indicating reduced selenocompound-based antioxidant defense. Ubiquitin-mediated proteolysis promotes cellular protein turnover by degrading abnormal proteins and maintaining cellular homeostasis.^66^ In this study, DNB genes were associated with enrichment of ubiquitin-mediated proteolysis. Notably, *tnks*, a key gene that encodes poly-ADP-ribosyltransferase, involved in telomere maintenance^67,68^ was identified as a down-regulated DNB gene. GO terms related to telomere maintenance were significantly enriched. These results imply that the maintenance of cellular homeostasis and normal telomere structure are markedly affected by death.

*Mhca*, a key gene that encodes Myosin-2, which is a critical protein for cell cycle and division,^69^ was identified as a target of bmo-miR-2795. This reveals the role of miRNAs in regulating cell cycle and division post-mortem. Octopamine receptors (key targets for the development of novel insecticides) and transposable elements help insects to respond and adapt to external stresses.^70,71^ In the current study, *oa1*, which encodes octopamine receptors, was identified as a target of bmo-miR-2762, whereas bmo-miR-1000 targeted *tcb1* gene, which encodes transposable element transposase. These 2 targets exhibited down-regulated expression in post-mortem silkworms compared to living ones, possibly indicating decreased resistance of silkworms caused by the suppression of miRNAs affecting neurotransmitters and hormones after death. Transcription factors are implicated in transcriptional regulation in animal post-mortem.^1^ However, their post-transcriptional regulation remains unclear. In this study, we observed that bmo-miR-2784 and bmo-miR-277-5p targeted 2 transcription factors, *achi* and *creb*, respectively, revealing for the first time the mechanism of post-transcriptional regulation of transcription factors post-mortem. Moreover, *achi*, a key member of TGF-beta signalling pathway, primarily involved in wound healing, tissue, and immune homeostasis,^72,73^ was upregulated in post-mortem silkworms, accompanied by down-regulated expression of bmo-miR-2784. This finding implies that homeostasis is maintained by alleviation of miRNA regulation post-mortem, further emphasizing the significance of homeostasis even after death.

In summary, we identified core post-mortem-related genes in silkworm, highlighting the crucial role of several genes in organisms after death. In addition, time-dependent kinetic changes in gene expression were observed post-mortem, revealing the mechanisms underlying the dynamic changes in global gene expression throughout the post-mortem process. Notably, a significant shift in global gene expression was observed at 2 h post-post-mortem in silkworms, unveiling the key genes involved in this pivotal event. Furthermore, this study is the first to reveal a comprehensive miRNA–mRNA regulatory network in post-mortem, providing key insights into the miRNA-mediated post-transcriptional regulation after death. However, further investigations using physiological and biochemical approaches are required to uncover the intricacies of post-mortem events in the future.

## Supplementary Material

dsae031_suppl_Supplementary_Figures

dsae031_suppl_Supplementary_Tables

## Data Availability

Clean reads of 40 sequencing libraries were submitted to the NCBI Sequence Read Archive (SRA) database under accession BioProject numbers: PRJNA718644 (transcriptomics) and PRJNA721793 (microRNAomics). These data are publicly available.
