# Supplementary material for: Time-dependent changes in genome-wide gene expression and post-transcriptional regulation across the post-death process in silkworm
Source: DNA Res. 2024 Nov 15;31(6):dsae031. doi: 10.1093/dnares/dsae031 (PMC11605879; doi:10.1093/dnares/dsae031)
Supplement: dsae031_suppl_Supplementary_Figures [file dsae031_suppl_supplementary_figures.doc]

**Supplementary Figures**


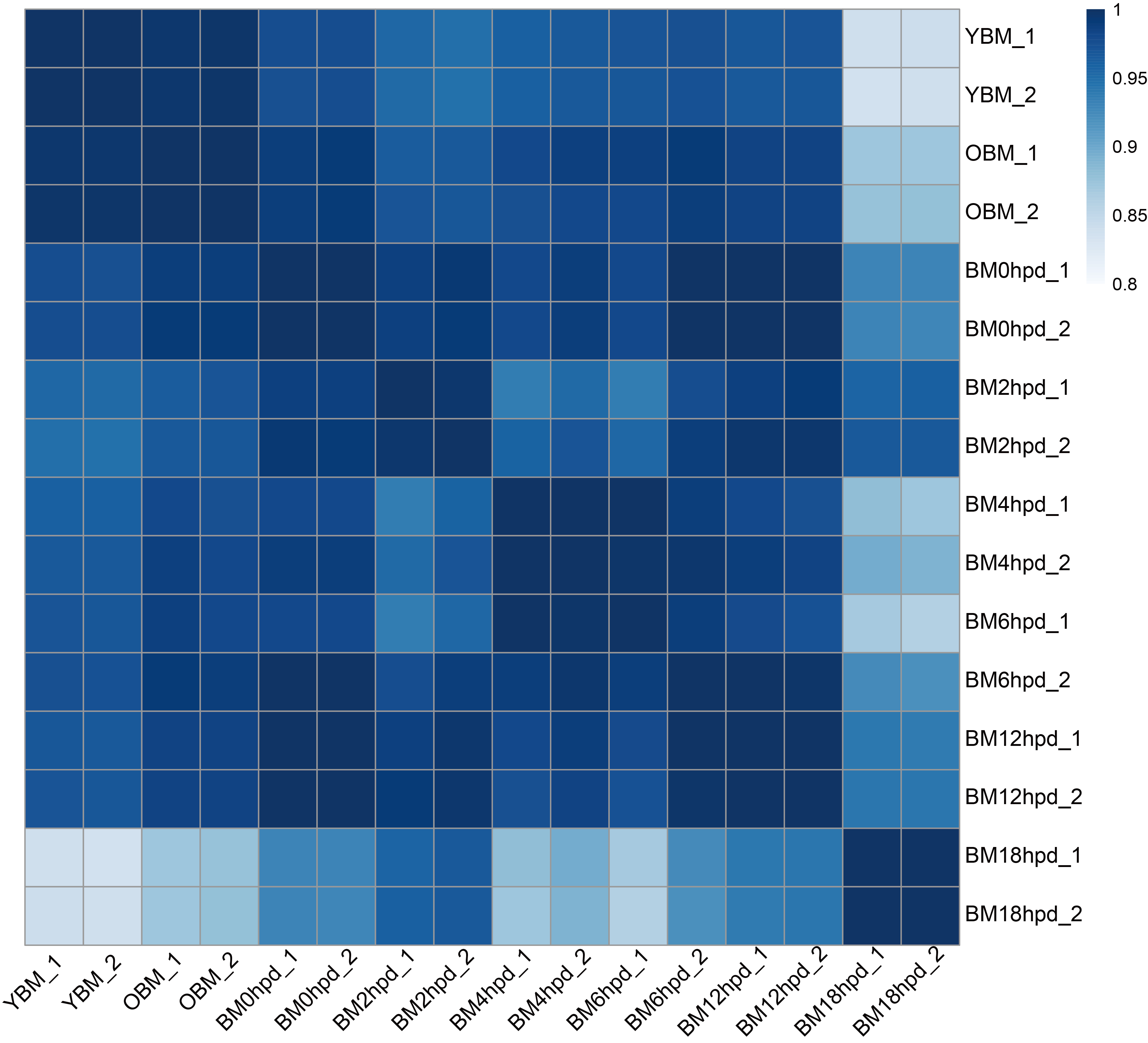


**Supplementary figure 1.** Correlation heat map among microRNAomes across eight time points.


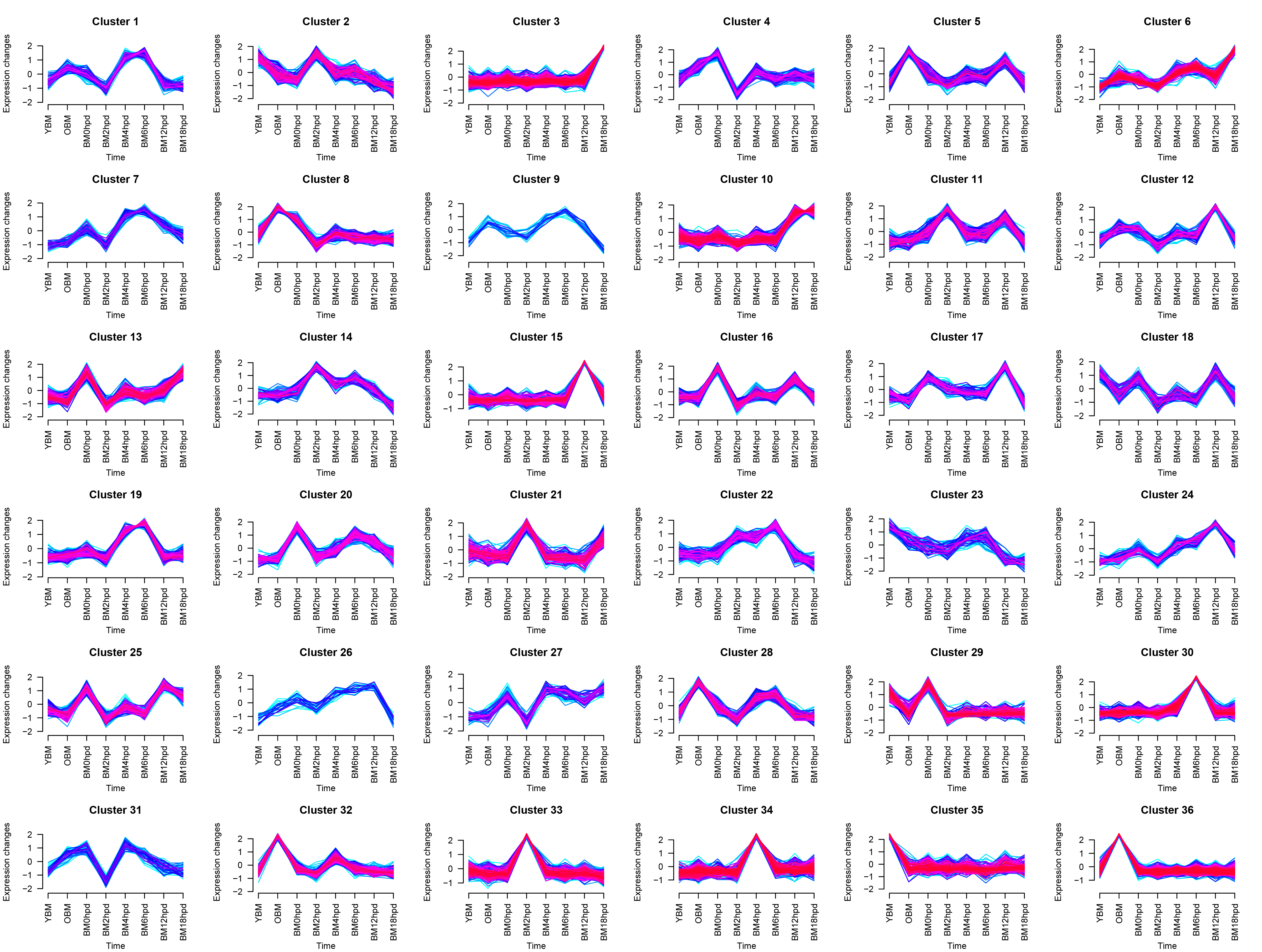


**Supplementary figure 2.** In total, all the genes were assigned to 36 gene expression clusters by Mfuzz analysis.


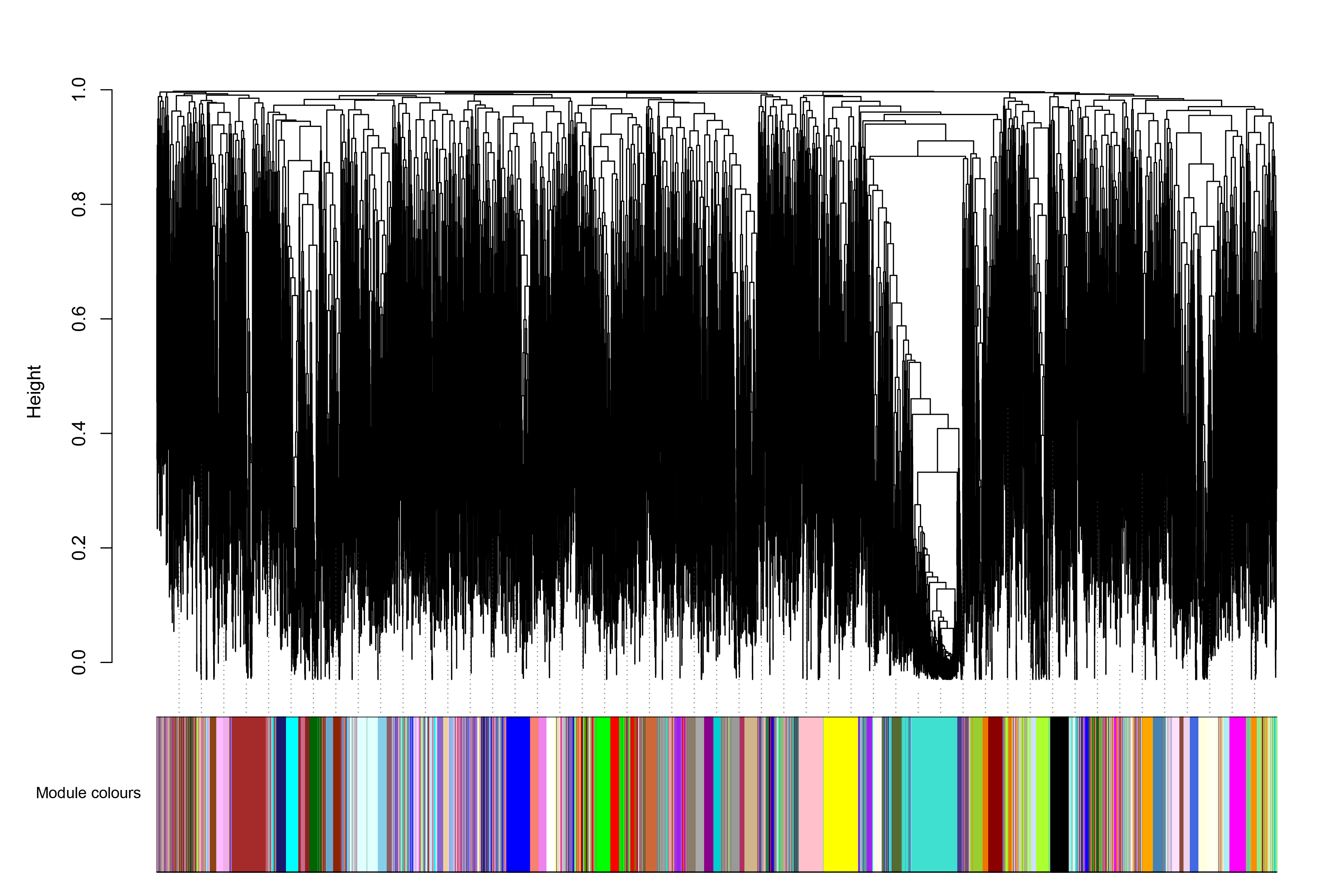


**Supplementary figure 3.** Gene modules identified by WGCNA for the combination of transcriptomics and microRNAomics data. Clustering dendrograms of genes, with dissimilarity based on topological overlap. Each branch represented a miRNA or mRNA, and each color below represented a co-expression module.

| **Abbreviation** | **Function descriptions** |
| --- | --- |
| *hsp1* | heat shock protein 1 |
| *il1b* | interleukin-1b |
| *tnpo1* | transportin 1 |
| *ter94* | transitional endoplasmic reticulum ATPase TER94 |
| *pin1* | rotamase Pin1 |
| *tdh* | L-threonine dehydrogenase |
| *acbd6l* | acyl-CoA-binding domain-containing protein 6-like |
| *LOC101736312* | uncharacterized LOC101736312 |
| *znf528* | zinc finger protein 528 |
| *agpat1* | 1-acyl-sn-glycerol-3-phosphate acyltransferase alpha |
| *syne1* | nesprin-1 |
| *gkap1* | G kinase-anchoring protein 1 |
| *fmr1l* | synaptic functional regulator FMR1-like |
| *ephexin-1* | uncharacterized LOC101739343 |
| *mars* | methionine--tRNA ligase |
| *ndufb11* | NADH dehydrogenase [ubiquinone] 1 beta subcomplex subunit 11 |
| *cul2* | cullin-2 |
| *twk-18* | TWiK family of potassium channels protein 18 |
| *tnks* | tankyrase |
| *pias3* | E3 SUMO-protein ligase PIAS3 |
| *rumta* | tRNA (uracil-5-)-methyltransferase homolog A |
| *klc* | kinesin light chain |
| *sft-6* | surfeit locus protein 6 homolog |
| *mlxipl* | carbohydrate-responsive element-binding protein |
| *mettl6* | methyltransferase-like protein 6 |
| *gmps* | GMP synthase [glutamine-hydrolyzing] |
| *t3dh* | probable hydroxyacid-oxoacid transhydrogenase |
| *dlgap5* | disks large-associated protein 5 |
| *LOC101744949* | uncharacterized LOC101744949 |
| *LOC101746000* | uncharacterized LOC101746000 |
| *ampd2* | AMP deaminase 2 |
| *rpn3* | probable 26S proteasome non-ATPase regulatory subunit 3 |
| *polz1* | DNA polymerase zeta catalytic subunit |
| *fusl* | RNA-binding protein fusilli-like |
| *LOC105841936* | uncharacterized LOC105841936 |
| *chinmo* | zinc finger protein chinmo |
| *LOC110384788* | uncharacterized LOC110384788 |
| *msantd3* | myb/SANT-like DNA-binding domain-containing protein 3 |
| *nadh6l* | NADH-ubiquinone oxidoreductase chain 6-like |
| *hsp90* | 90-kDa heat shock protein |
| *desat1* | acyl-CoA delta-11 desaturase/conjugase |
| *wh3* | ATP dependent transmembrane transporter protein |
| *vgr* | vitellogenin receptor |
| *plscr* | phospholipid scramblase |
| *cec1* | cecropin A |
| *cpfl4* | cuticular protein CPFL family 4 |
| *cpr78* | cuticular protein RR-2 motif 78 |
| *hsp68* | heat shock protein 68 |
| *LOC101735378* | uncharacterized LOC101735378 |
| *tug* | tether containing UBX domain for GLUT4 |
| *tpgs2* | tubulin polyglutamylase complex subunit 2 |
| *crn* | atrial natriuretic peptide-converting enzyme |
| *kif19* | kinesin-like protein KIF19 |
| *nalcn* | sodium leak channel non-selective protein |
| *prp4k* | serine/threonine-protein kinase PRP4 homolog |
| *cfap251* | cilia- and flagella-associated protein 251 |
| *tgmh1* | hemocyte protein-glutamine gamma-glutamyltransferase |
| *cyp4c21* | cytochrome P450 4c21 |
| *LOC101738342* | uncharacterized LOC101738342 |
| *sf3b1* | splicing factor 3B subunit 3 |
| *pdzd8* | PDZ domain-containing protein 8 |
| *me1* | NADP-dependent malic enzyme |
| *traf3ip1* | TRAF3-interacting protein 1 |
| *iqcg* | IQ domain-containing protein G |
| *LOC101739801* | uncharacterized LOC101739801 |
| *spty2d1* | protein SPT2 homolog |
| *myo7* | Myo7 |
| *ext2* | Extensin-2 |
| *ndu1* | NADH dehydrogenase (ubiquinone) complex I |
| *chym2* | Chymotrypsin-2 |
| *sp5* | transcription factor Sp5 |
| *LOC101741846* | uncharacterized LOC101741846 |
| *LOC101741944* | uncharacterized LOC101741944 |
| *LOC101742185* | uncharacterized LOC101742185 |
| *fbl* | rRNA 2'-O-methyltransferase fibrillarin |
| *rnf212* | probable E3 SUMO-protein ligase RNF212 |
| *LOC101742559* | uncharacterized LOC101742559 |
| *ox2* | homeobox protein 2 |
| *LOC101742779* | uncharacterized LOC101742779 |
| *LOC101742906* | uncharacterized LOC101742906 |
| *cec1l* | cecropin-A-like |
| *LOC101743622* | putative uncharacterized protein DDB_G0282499 |
| *mettl6* | methyltransferase-like protein 6 |
| *cart* | carcinine transporter |
| *LOC101743946* | uncharacterized LOC101743946 |
| *msh-a* | homeobox protein MSH-A |
| *chym1* | chymotrypsin-1 |
| *LOC101744334* | uncharacterized LOC101744334 |
| *smpd1* | sphingomyelin phosphodiesterase 1 |
| *spag6* | sperm-associated antigen 6 |
| *hsp70* | heat shock 70 kDa protein |
| *dnah12* | dynein heavy chain 12 |
| *rpc8* | DNA-directed RNA polymerase III subunit RPC8 |
| *LOC101746357* | uncharacterized LOC101746357 |
| *LOC101746909* | uncharacterized LOC101746909 |
| *LOC105841476* | uncharacterized LOC105841476 |
| *LOC105842407* | uncharacterized LOC105842407 |
| *LOC105842505* | uncharacterized LOC105842505 |
| *LOC105842971* | uncharacterized LOC105842971 |
| *LOC110384712* | uncharacterized LOC110384712 |
| *lap3l* | cytosol aminopeptidase-like |
| *lip3l* | lipase 3-like |
| *LOC110385013* | uncharacterized LOC110385013 |
| *fhxl* | fibrohexamerin-like |
| *cbp* | carotenoid-binding protein |
| *nachrb3* | nicotinic acetylcholine receptor subunit beta 3 |
| *cpr23* | cuticular protein RR-1 motif 23 |
| *cce6* | carboxyl/cholinesterase 6 |
| *rdh11l* | retinol dehydrogenase 11-like |
| *tll1* | tolloid-like protein 1 |
| *atl* | atlastin |
| *lalrap1* | low density lipoprotein receptor adapter protein 1 |
| *tfkc* | triokinase/FMN cyclase |
| *LOC101738364* | uncharacterized LOC101738364 |
| *LOC101740628* | uncharacterized LOC101740628 |
| *agpat5* | 1-acylglycerol-3-phosphate O-acyltransferase 5 |
| *scp* | sarcoplasmic calcium-binding protein |
| *LOC101741814* | uncharacterized LOC101741814 |
| *slc25a29* | mitochondrial basic amino acids transporter |
| *LOC101745048* | uncharacterized LOC101745048 |
| *mala3l* | maltase A3-like |
| *oct* | organic cation transporter protein-like |
| *cpamd8* | C3 and PZP-like alpha-2-macroglobulin domain-containing protein 8 |
| *nfib* | nuclear factor 1 B-type |
| *LOC105841590* | uncharacterized LOC105841590 |
| *serac1l* | protein SERAC1-like |
| *smctn* | sodium-coupled monocarboxylate transporter 2 |
| *atp8a1* | probable phospholipid-transporting ATPase IA |
| *pyx* | transient receptor potential channel pyrexia |
| *c11orf54* | ester hydrolase C11orf54 homolog |
| *hao1* | hydroxyacid oxidase 1 |
| *nox4* | NADPH oxidase 4 |
| *cyp4g15l* | cytochrome P450 4g15-like |
| *lrrc40* | leucine-rich repeat-containing protein 40 |
| *cyp4g15l* | cytochrome P450 4g15 |
| *cryz* | quinone oxidoreductase |
| *itm23* | 23 kDa integral membrane protein |
| *sod* | superoxide dismutase [Cu-Zn] |
| *LOC101744704* | uncharacterized LOC101744704 |
| *rnz* | ribonuclease Z |
| *LOC101744376* | uncharacterized LOC101744376 |
| *try-1* | trypsin-1 |
| *LOC101743981* | uncharacterized LOC101743981 |
| *obst-e* | protein obstructor-E |
| *agt2l* | alanine--glyoxylate aminotransferase 2-like |
| *acss3* | acyl-CoA synthetase short-chain family member 3 |
| *plscr2* | phospholipid scramblase 2 |
| *kcnj2l* | inward rectifier potassium channel 2-like |
| *mtf* | mitochondrial folate transporter/carrier |
| *myom* | myosin-M heavy chain |
| *LOC101742870* | uncharacterized LOC101742870 |
| *apepp* | xaa-Pro aminopeptidase ApepP |
| *LOC101741820* | uncharacterized LOC101741820 |
| *micu1* | calcium uptake protein 1 homolog |
| *celf1* | CUGBP Elav-like family member 1 |
| *slc9a3* | sodium/hydrogen exchanger 3 |
| *tws* | protein phosphatase PP2A 55 kDa regulatory subunit |
| *LOC101740514* | uncharacterized LOC101740514 |
| *bbp* | branchpoint-bridging protein |
| *abca1* | ATP-binding cassette sub-family A member 1 |
| *fas* | fatty acid synthase |
| *cg1544* | probable 2-oxoglutarate dehydrogenase E1 component DHKTD1 homolog |
| *twk-18* | TWiK family of potassium channels protein 18 |
| *ppp2r3d* | serine/threonine-protein phosphatase 2A regulatory subunit B'' subunit delta |
| *LOC101739653* | uncharacterized LOC101739653 |
| *nup85* | nuclear pore complex protein Nup85 |
| *sdhfp* | succinate dehydrogenase [ubiquinone] flavoprotein subunit |
| *let-4* | leucine-rich repeat-containing protein let-4 |
| *beta-1* | beta-1 |
| *acaa* | 3-ketoacyl-CoA thiolase |
| *LOC101738136* | uncharacterized LOC101738136 |
| *LOC101738091* | uncharacterized LOC101738091 |
| *pclo* | protein piccolo |
| *lrrc24* | leucine-rich repeat-containing protein 24 |
| *mpv17* | protein Mpv17 |
| *LOC101737750* | uncharacterized LOC101737750 |
| *chad* | chondroadherin |
| *pcft* | proton-coupled folate transporter |
| *srap* | serine-rich adhesin for platelets |
| *mvg* | microvitellogenin |
| *klf10* | Krueppel-like factor 10 |
| *neurochondrin* | neurochondrin homolog |
| *lrrn3* | leucine-rich repeat neuronal protein 3 |
| *mrpl11* | 39S ribosomal protein L11 |
| *k12h4.7* | putative serine protease K12H4.7 |
| *zig-8* | zwei Ig domain protein zig-8 |
| *sh3rf1* | E3 ubiquitin-protein ligase SH3RF1 |
| *LOC101735793* | uncharacterized LOC101735793 |
| *mdr49* | multidrug resistance protein homolog 49 |
| *myph* | myophilin |
| *pinta* | retinol-binding protein pinta |
| *abc* | ABC transporter |
| *apa* | apterous A |
| *bm8ip2d-4* | Bm8 interacting protein 2d-4 |
| *cph11* | cuticular protein hypothetical 11 |
| *cpr5* | cuticular protein RR-1 motif 5 |
| *ibm1* | IAP-binding motif 1 |
| *gps73bl* | G protein alpha subunit 73B-like protein |
| *ubx* | ultrabithorax |
| *abd-a* | abdominal A |
| *cyp* | cytochrome P450 |
| *obp2* | general odorant binding protein 2 |
| *obp1* | general odorant binding protein 1 |
| *pbp* | pheromone binding protein |
| *p270* | p270 |
| *apc11* | anaphase promoting complex subunit 11 |
| *gstd1* | glutathione S-transferase delta 1 |
| *cpr46* | cuticular protein RR-1 motif 46 |
| *or3* | olfactory receptor 3 |
| *cpg4* | cuticular protein glycine-rich 4 |
